# Obtaining help by standing higher: The mediating role of moral reputation

**DOI:** 10.3389/fpsyg.2022.1013656

**Published:** 2022-12-22

**Authors:** Xiaodong Ming, Jingyu Fu, Jianfeng Yang

**Affiliations:** ^1^School of Business Administration, Jiangxi University of Finance and Economics, Nanchang, Jiangxi Province, China; ^2^Business School, University of International Business and Economics, Beijing, China

**Keywords:** vertical spatial position, helping behavior, prosocial behavior, conceptual metaphor, moral reputation

## Abstract

Drawing on the social cognitive chain of being (SCCB) theory and heuristic perspective, the present study explored whether and how social targets’ vertical spatial position influences the help the social targets can get from others. Study 1 demonstrated that individuals would be more likely to help social targets who were presented on a higher vertical spatial position than those who were presented on a lower vertical spatial position. In Study 2, an experimental-causal-chain design was adopted for further testing the mediating role of moral reputation between the social targets’ vertical spatial position and the amount of help that the social targets obtain from others. Study 3 cross-validated this mediating process by a measurement-of-mediation design. Those three studies help us comprehend how helping behavior occurs from the characteristics of help recipients as well as extend the influence of vertical spatial metaphor of morality from cognitive connection to action-relevant outcomes.

## 1. Introduction

Prosocial behavior refers to voluntary action that is intended to benefit others, including various actions such as helping, cooperating, and caring (Penner et al., 2005). Prosocial behavior has been found to be beneficial to all aspects of our society (Laguna et al., 2020). For example, prosocial behavior can promote the romantic partners’ health and wellbeing (Impett et al., 2015), enhance organizational productivity (Podsakoff et al., 2014) as well as reduce poverty of the whole society (Böhm et al., 2018). With so many important benefits, prosocial behavior has captured the attention of multiple disciplines, including biology, psychology, economics, and sociology (Penner et al., 2005).

Previous studies made a lot of efforts to explore the antecedents of prosocial behavior at three levels, i.e., the micro level, the meso level, and the macro level (Penner et al., 2005). Antecedents at the micro-level looked into the origins of prosocial tendencies and the sources of variation in these tendencies (Penner et al., 2005). At the micro-level, for example, helpers’ personality has long been viewed as an important antecedent in influencing prosocial behavior (Thielmann et al., 2020). Meso-level involves helper-recipient dyads in the context of a specific situation (Penner et al., 2005). At the meso-level, for example, potential rewards of helping could enhance helpers’ prosocial behavior, while potential punishments of helping could lower helpers’ prosocial behavior (Wu et al., 2022). The macro-level antecedents involve prosocial actions that occur within the context of groups and large organizations (Penner et al., 2005). At the macro-level, for example, collectivism could enhance prosocial behavior (Lee and Kim, 2021). While previous studies at these three levels have examined many antecedents that could influence prosocial behavior, these studies may ignore the recipients’ factors that may influence the prosocial action of the helper (Wang et al., 2018). In the helper-recipient dyads, prior studies tended to regard the recipients as a passive side of prosocial action and as having nothing to do with the occurrence of prosocial behavior (Bamberger, 2009; Thompson and Bolino, 2018). However, this may generate a misunderstanding regarding the occurrence of prosocial behavior.

Based on the social cognitive chain of being theory (SCCB; Brandt and Reyna, 2011) and heuristic perspective (Tversky and Kahneman, 1974), this study attempted to explore whether a help recipient’s vertical spatial position, as a recipients’ factor, can influence others’ helping behavior towards the recipient through the meditation role of the recipient moral reputation. The SCCB theory claims that perceptions of morality are on a vertical continuum, and the spatial vertical position and the morality are two strongly related dimensions (Brandt and Reyna, 2011). Meanwhile, according to the heuristic perspective, individuals can infer about the related dimension of unclear information using one dimension of clear information (Qin et al., 2015). Thus, help recipients’ unclear moral reputation can be inferred from help recipients’ vertical spatial position by the helper. In addition, the SCCB theory also suggests that the moral person who is on the higher chain of being would obtain more help from others (Brandt and Reyna, 2011).

The present study contributes to the literature of prosocial behavior and conceptual metaphor in several ways. Firstly, this study explored the antecedents of prosocial behavior in terms of the recipient’s characteristics, which could deepen our understanding of the reason why prosocial behavior occurs. Prior studies mostly regarded recipients as the passive side of prosocial behavior, neglecting help recipients’ influences on the occurrence of prosocial behavior (Bamberger, 2009; Thompson and Bolino, 2018). In this study, we explored how the help recipients’ spatial vertical position function in activating the helper’s prosocial behavior towards the help recipients, which has broadened the antecedents of prosocial behavior.

Secondly, this study helps to clarify the relationship between vertical spatial metaphor and moral cognition. Most prior studies imply that morality will prime spatial vertical metaphor, that is to say, “moral is up/immoral is down” (Li and Cao, 2017). However, according to the heuristic perspective, it can be argued that the spatial vertical information will also prime moral cognition, which would deepen our understanding of the relationship between vertical spatial metaphor and moral cognition.

Thirdly, this study would extend the effects of the vertical spatial metaphor of morality from cognitive connection to action-relevant outcomes. The cognitive connection between vertical spatial metaphor and moral cognition has been supported by numerous studies (e.g., Meier and Robinson, 2004; Meier et al., 2007a; Hill and Lapsley, 2009). However, few studies have explored the action-relevant outcomes of the vertical spatial metaphor, which deserve further exploration (Meier et al., 2012).

Finally, the findings of this study can also contribute to the SCCB theory. The SCCB theory claims that the social targets’ location on the chain of being is consequential and dynamic. The SCCB theory argues that the social targets’ position on the chain of being is influenced by emotional cues (e.g., disgust, shame, elevation) and motivations (e.g., self-and group-serving motivation, effectance motivation, and existential motivations). However, the current study examined the more overt information of the social target, the spatial vertical position, which can also influence the social targets’ position on the chain of being.

## 2. Theories and hypotheses

### 2.1. The social cognitive chain of being theory

The SCCB theory is a framework concerning the moral cognition of humans. The theory supposes that people use a specific position in a vertical chain to represent a social target’s moral status and adjust their behavior towards the target according to its vertical position in the chain (Brandt and Reyna, 2011).

According to the SCCB theory, morality is difficult for individuals to understand; therefore, individuals tend to use concrete concepts such as “up” and “down” to understand the abstract concept of morality. Individuals would even employ a social cognitive chain to denote social target’s morality. At the highest target of the chain is God, and the at lowest level is Satan, each representing the ultimate morality and immorality, respectively. There exists saint, human, animal, and so on between the two poles. A social target closer to the up pole (God) is perceived as more ethical (e.g., saint); and a social target closer to the down pole (Satan) is perceived as more unethical (e.g., animal) (Brandt and Reyna, 2011). At the same time, the positions of targets in the chain would influence others’ attitudes and behavior towards the targets, including rejection or acceptance (Brandt and Reyna, 2011).

Moreover, the social targets’ location in the chain of being is consequential and dynamic (Brandt and Reyna, 2011). What influence social targets’ location in the chain of being are emotional cues and motivation. In terms of emotional cues, moral emotions such as awe, elevation, pride, and self-satisfaction could cue someone at a higher position of the chain of being, whereas contempt, disgust, shame, and guilt may cue someone at a lower position of the chain of being. In terms of motivations, self-and group-serving motivation may lead individuals to put themselves or ingroup individuals at a higher position of the chain of being, and to put others or outgroups at a lower position of the chain of being. Effectance motivation impel individuals to control the uncertainty, thus individuals will use deity anthropomorphism and dehumanization to put social targets on a lower position on the chain of being. Individuals may also use animalistic anthropomorphism and sanctification to put social targets in a higher position of the chain of being. In addition, existential motivation leads individuals to put themselves at a higher position along the chain of being to pursue the ultimate personal meaning and purpose in life.

### 2.2. Social targets’ vertical spatial position and their moral reputation

Individuals would apply vertical spatial concepts (e.g., up and down) to express the abstract concept of morality (Brandt and Reyna, 2011). For human beings, early life experience is important to shape the contact between “up” (“down”) and morality (immorality) (Fiske, 2004). In the infancy period, the guardian is usually at the upside of an infant’s scope of vision, which forms a linkage between a high-position target and love or morality in the infant’s mind (Fiske, 2004). Moral emotions, including shame and embarrassment, imply that one may have violated a personal or social norm, which can be characterized by positioning the head and eye gaze downward in different cultures (e.g., Keltner, 1995; Tracy and Matsumoto, 2008). These early life experiences provide concrete physical experiences that are necessarily required to understand the more complex and higher mental processes of moral perception (Brandt and Reyna, 2011).

Furthermore, the vertical spatial relationship is the most basic spatial relationship since it is rooted in the earth’s gravity; therefore, it’s one of the most direct and profound spatial relationships for human beings (Gibson, 1969). The link between vertical spatial position and morality exists at both psychological and linguistic levels, as demonstrated by constructions represented by “high-minded,” “on the up and up,” “down and dirty,” “low-minded,” and “underhand” (Lakoff and Johnson, 1999). As the above terms implied, morality is usually connected with “high” or “up” and immorality with “down,” “low,” or “under.” This linkage can help human beings use the specific concept of “high and low” to comprehend abstract concepts that could not be understood directly, like “morality” (Meier et al., 2007a; Lin and Oyserman, 2021).

Empirical findings have also supported the implicit association between the verticality metaphor and morality/immorality. Words associated with morality have been shown to have a stronger link with being up than with being down. Likewise, immorality-related words have a stronger link with being down than with being up (Meier et al., 2007a,b; Wang et al., 2016). Individuals are inclined to infer unclear information concerning a dimension based on a related dimension with clear information (Qin et al., 2015). For this reason, when the morality information of a person is unclear, individuals could infer the person’s moral information based on the related vertical spatial position. Thus, we hypothesize the following:

*Hypothesis 1*: Social targets’ vertical spatial position is positively related to the social targets’ moral reputation, such that social targets at a higher vertical position would be judged to be more ethical than those at a lower vertical position.

### 2.3. Social targets’ moral reputation and others’ helping behavior towards them

One important viewpoint of SCCB claims that the social targets’ location in the chain of being is consequential and dynamic (Brandt and Reyna, 2011). As the perception of social targets moves down along the chain of being, from being divine to being devilish, the social targets will be perceived to be more immoral and worthy of blame, exclusion, and discrimination. Under the circumstances, the perceiver no longer believes the target is worthy of care and concern (Brandt and Reyna, 2011). Conversely, as the perception of social targets moves up along the chain of being, from being devilish to being divine, the social targets will be perceived to be more ethical and worthy of support, care, and concern as well as protection against secular encroachments (Brandt and Reyna, 2011).

On one hand, the social target will experience a process of dehumanization if it is perceived as less ethical by individuals (Brandt and Reyna, 2011). The target is thus considered less valuable than a human being, which does not merit kind treatment. For example, the targets will be excluded from their moral universe if they are perceived as less moral than human beings (Opotow, 2001), while receiving less compassion and empathy from others (Bandura, 2002).

On the other hand, a process of sanctification will occur to the targets who are perceived to be more ethical, and these social targets receive more positive treatment from others. Although there is a lack of direct empirical evidence to support this proposition, some indirect evidence indeed exists. For example, individuals who sanctify their marriages will have stable marriages (Mahoney et al., 2003). Individuals who sanctify the environment are more likely to donate for environmental protection (Tarakeshwar et al., 2001), and individuals who sanctify parenting are more committed to the value of parenting (Murray-Swank et al., 2006).

In sum, individuals hold that a social target at a lower position of the chain of being does not deserve the same moral consideration compared to that at a higher position in the chain, while a social target at a higher position of the chain of being deserves more moral consideration compared to that at a lower position in the chain (Brandt and Reyna, 2011). Thus, we hypothesize the following:

*Hypothesis 2*: Compared with immoral social targets, moral social targets will get more help from others.

In the prior sections, we posit that social targets at a higher vertical spatial position will be judged as more ethical than those at a lower vertical spatial position. In general, compared with lower-position social targets, higher-position ones are more likely to be judged as moral people, which may result in a higher location in the chain of being and further affect how much help those people can get from others. Thus, we hypothesize the following:

*Hypothesis 3*: Social targets at a higher vertical spatial position are inclined to get more help than those at a lower vertical spatial position.

*Hypothesis 4*: Moral reputation mediates the positive relationship between social targets’ vertical spatial position and the helping behavior that they received.

## 3. Overview of the present study

In Study 1, we examined whether social targets’ vertical spatial position was positively related to others’ helping behavior towards the social targets. On this basis, we predicted that social targets at a high vertical spatial position would get more help than those at a low vertical spatial position. In Study 2, an experimental-causal-chain design (Spencer et al., 2005) was used to explore the mediating role of social targets’ moral reputation between their vertical spatial position and the help received from others. Study 2a explored the relationship between social targets’ vertical spatial position and their moral reputation. According to the results, it was predicted that social targets at a high vertical spatial position would be judged as more moral than those at a low vertical spatial position. In Study 2b, we directly manipulated a hypothetical social target’s moral reputation to investigate the relationship between social targets’ moral reputation and the help they received from others. In the meantime, moral targets were expected to get more help from others than immoral targets. In Study 3, the measurement-of-mediation design (Baron and Kenny, 1986) was adopted to cross-validate the mediating role of moral reputation between vertical spatial position and helping behavior.

## 4. Study 1

### 4.1. Participants

Power analysis needs a starting point. Our best guess of effect size came from Cohen’s *d* = 0.94 reported in Wang et al. (2016). By adopting the G*Power program (Faul et al., 2007), we calculated the necessary samples to do an experiment of a single-factor design with two levels (Effect Size *d* = 0.94, Type I Error = 0.05, Statistical Power = 0.80). The recommended sample size in each group is 19. Accordingly, 70 undergraduates from a university in southern China (12 males, 58 females; *M_age_* = 20.07, *SD* = 0.74) were recruited to participate in the current experiment. They all completed the experiment in the same laboratory.

### 4.2. Procedures

**Figure 1:**
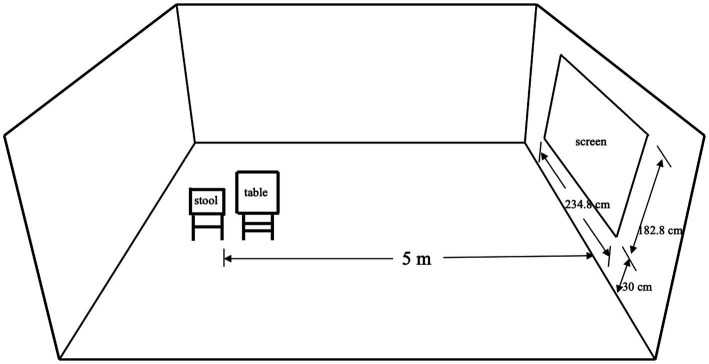
A schematic diagram of the lab.

The experiment is a single-factor design with two levels (high position vs. low position). The participants were randomly assigned to the low position or high position condition and entered the laboratory one by one. Then, the experimental materials appeared on the laboratory’s projection screen (234.8 cm*182.8 cm), telling the participants that two young faculty members in our school were looking for volunteers to help them with some research work. The volunteer’s job description included collecting experimental materials, inputting data, and so on. These tasks were done on a voluntary basis without any pay. We presented these details and two white background photos of the faculty members’ front-facing heads and shoulders on the screen (one male and one female, each photo with a size of 34 cm*53 cm, both faculties with a neutral expression, and these two photos are abreast on the middle part of the screen). One group of participants saw the photos of the faculty members on the upper part of the screen, while the other group of participants saw the photos on the lower part of the screen. The descriptive information remained the same in these two conditions. Sitting on a stool 5 meters away from the screen, the participant started to read the information on the screen. The schematic diagram of the lab is shown in Figure 1. Then the participants were asked to write down their answers to questions about whether they would like to help those two faculty members (yes/no) and how much time they would like to spend (hours) if they were willing to assist. They were also asked to provide their telephone number to be informed of the details of their voluntary work. After finishing these processes, the researcher debriefed the participants. No participant correctly guessed the true objective of this study.

### 4.3. Results and discussion

**Figure 2:**
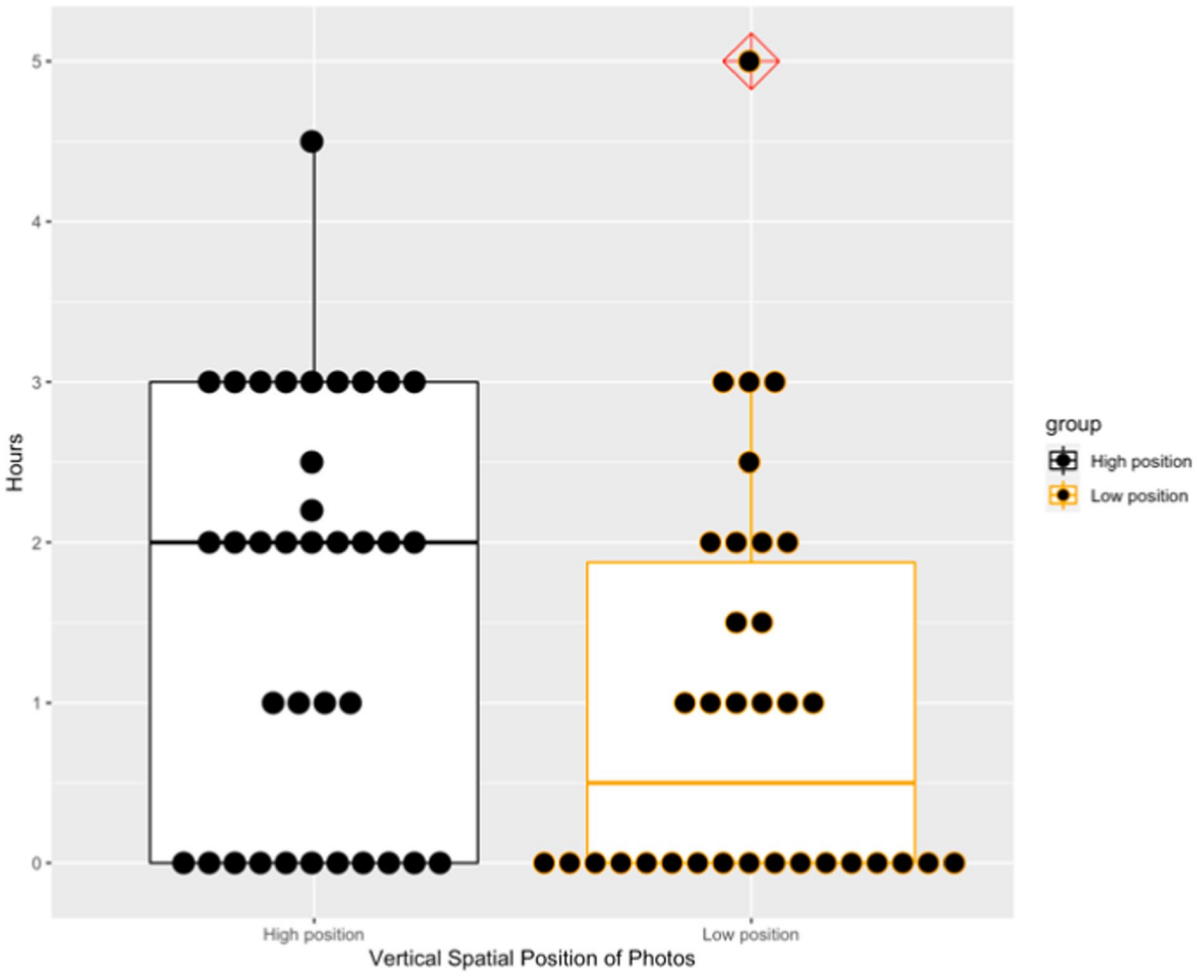
Two groups’ helping hours in Study 1.

Although there only existed a marginal difference in intention to help between the high and low position conditions (69.44% vs. 50%, *χ*^2^ = 2.76, *df* = 1, *p* = 0.097), a significant difference was observed in the length of time that the participants were willing to provide (*t* = 2.08, *p* = 0.042, Cohen’s *d* = 0.50). As predicted, the participants offered more time to help the faculty members when the photos were displayed on the upper part of the screen (*M* = 1.62, *SD* = 1.29) than when the photos were displayed on the bottom of the screen (*M* = 0.99, *SD* = 1.25). Figure 2 presents the boxplot of the two groups’ helping hours. In consistence with *Hypothesis* 3, the faculties’ vertical spatial position was positively related to others’ helping behavior toward them. Besides, the faculty member on high position tended to get more help than the faculty member on low position. In Study 2, we further explored the mediating role of moral reputation between social targets’ vertical spatial position and others’ helping behavior toward them.

## 5. Study 2

### 5.1. Study 2a

#### 5.1.1. Participants

Just like Study 1, our best guess of effect size came from Cohen’s *d* = 0.94 reported in Wang et al. (2016). By using the G*Power (Faul et al., 2007), we calculated the necessary samples to perform an experiment of a single-factor design with two levels (Effect Size *d* = 0.94, Type I Error = 0.05, Statistical Power = 0.80). The recommended sample size in each group is 19. Accordingly, a total of 68 undergraduates from a university in southern China (28 male, 40 female, *M_age_* = 19.47, *SD* = 1.09) were recruited to participate in the current experiment. They all completed the experiment in a laboratory.

#### 5.1.2. Procedures

The experiment is a single-factor design with two levels (low position vs. high position). The 68 participants were randomly assigned to one of two conditions and entered the laboratory one by one. In addition, the participants were told that the researchers were investigators from the School of Business Administration and had been commissioned by the Dean of the school to investigate students’ first impressions of new faculty members, aiming to help the school establish a behavioral database of new faculty members. The resulting data can be used as a basis used for future recruitment of faculty members. Then, the researchers distributed questionnaire to the participant. The questionnaire contained measures of faculty’s morality, power, and the participants’ emotion. Next, the researchers projected two photos (one for each faculty) and a basic introduction to the two faculty members on a big screen in the laboratory. The order was counterbalanced based on the faculty’ sex in the photo. The lab and photos are the same as in Study 1. The descriptions were consistent across the two conditions. The only difference referred to was that the participants at the high condition saw the two photos on the upper part of the screen, while the participants at the low condition saw the two photos on the lower part of the screen. After reading the information displayed on the screen, the participants filled in the questionnaires. Finally, the researchers debriefed the participants. No participant guessed the true objective of the present study accurately.

#### 5.1.3. The moral reputation of the faculty members

The participants were invited to answer the following three questions on a nine-point scale from 1 (very immoral/unkind/dishonest) to 9 (very moral/kind/honest): (1) “What do you think of the female/male faculty’s moral level in the photos?” (2) “What do you think of the female/male faculty’s kindness level in the photos?” and (3) “What do you think of the female/male faculty’s honesty level in the photos?” The scale exhibited good reliability for both the female (*α* = 0.80) and male faculty (*α* = 0.85).

#### 5.1.4. Power of the faculty members

The faculty members’ power was measured with two items rated on a nine-point scale, from 1 (not at all) to 9 (very much): “How effectively do you think the female/male faculty in the photo can make the students obedient?” and “How much authority does the female/male faculty in the photo have?”

#### 5.1.5. Participants’ emotion

The participants indicated how they felt by answering the 20-item Positive and Negative Affectivity Scale (PANAS; Watson et al., 1988). The PANAS captured both positive affect (*α* = 0.87) and negative affect (*α* = 0.90) on a five-point scale (1 = very slightly or not at all, 5 = extremely).

#### 5.1.6. Results and discussion

**Figure 3:**
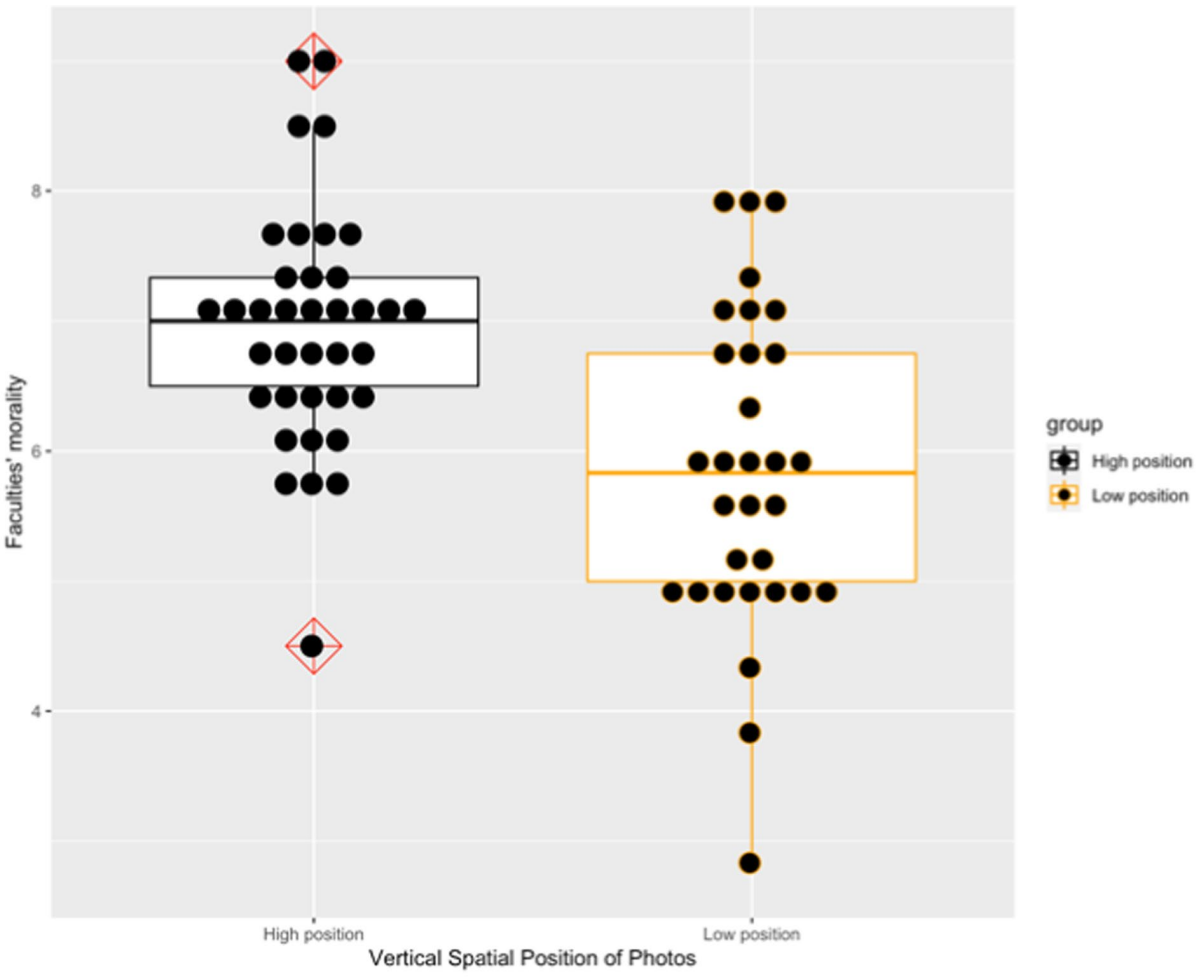
Two groups’ rating about the faculty members’ morality in Study 2a.

The male faculty was rated as more moral (*t* = 4.09, *p* < 0.001, Cohens’ *d* = 0.97) when his photo was projected on the upper part of the screen (*M* = 6.79, *SD* = 1.03) rather than the lower part of the screen (*M* = 5.59, *SD* = 1.41). Similarly, the female faculty was rated as more moral (*t* = 3.95, *p* < 0.001, Cohens’ *d* = 0.99) when her photo was displayed on the upper part of the screen (*M* = 7.10, *SD* = 0.93) rather than on the lower part of the screen (*M* = 6.09, *SD* = 1.12). By averaging each participant’s rating of the male faculty and the female faculty, the photos on the upper part of the screen (*M* = 6.95, *SD* = 0.92) were rated as significantly more moral (*t* = 4.29, *p* < 0.001, Cohens’ *d* = 1.03) in comparison with those on the lower part of the screen (*M* = 5.84, *SD* = 1.21). Figure 3 presents the boxplot of these two groups’ ratings about the faculty members’ morality.

However, there existed no significant difference in perceived power between the high condition and low condition for both the male faculty (*t* = 0.03, *p* = 0.977, Cohens’ *d* = 0.01) and the female faculty (*t* = 1.72, *p* = 0.090, Cohens’ *d* = 0.42). By averaging each participant’s rating of the male faculty and the female faculty, no significant differences (*t* = 0.95, *p* = 0.345, Cohens’ *d* = 0.23) were found in the perceived faculty members’ power. Furthermore, no significant difference was observed in positive affective scores (*t* = 1.02, *p* = 0.313, Cohens’ *d* = 0.25) and negative affective scores (*t* = 1.64, *p* = 0.106, Cohens’ *d* = 0.39) between the two experimental conditions. Generally, the obtained results support *Hypothesis 1* that social targets on a higher vertical spatial position are judged to be more moral than social targets on a lower vertical spatial position.

### 5.2. Study 2b

#### 5.2.1. Participants

Our best guess of effect size came from Cohen’s *d* = 0.81 reported in Wang et al. (2018). Using the G*Power (Faul et al., 2007), we calculated the necessary samples to conduct an experiment of a single factor-design with two levels (Effect Size *d* = 0.81, Type I error = 0.05, Statistical Power = 0.80). The recommended sample size in each group is 25. Accordingly, 71 undergraduates from a university in southern China (39 males, 30 females, 2 participants did not report gender, *M_age_* = 21.56, *SD* = 1.09) were recruited to participate in the current experiment. Besides, they completed the experiment in the same laboratory.

#### 5.2.2. Procedures

The experiment is a single-factor design with two levels. The participants were randomly assigned to one of two conditions (high moral reputation vs. low moral reputation). After becoming settled in the laboratory, the participants were informed that the study was about undergraduates’ emotions and decision-making. The researchers gave each participant two questionnaires and asked them to answer the questions in order. In both conditions, the participants were given a description of Xiao Ming, an alumnus of the participants’ university, who was seriously ill and in need of money for medical expenses. Subsequently, the participants were asked to write down whether they wanted to donate money to this alumnus. The moral reputation of the alumnus was the only difference between the two conditions. In the moral condition of reputation, the alumnus in need was described as a moral model, whereas in the immoral condition of reputation, this alumnus was depicted as having a habit of stealing. Afterwards, the participants indicated their emotion by answering the 20 items of the PANAS (Watson et al., 1988) on a 5-point scale (1 = very slightly or not at all, 5 = extremely), revealing high reliability for both positive affect (*α* = 0.89) and negative affect (*α* = 0.91). Apart from that, the participants were asked to use a nine-point scale (1 = not at all, 9 = very much) to rate the alumnus’ moral reputation (*α* = 0.96) by answering the following questions: (1) “How do you rate Xiao Ming’s moral level?” (2) “How do you rate the kindness of Xiao Ming?” and (3) “How do you rate Xiao Ming’s honesty?” Finally, the researchers debriefed the participants. No participant accurately guessed the true objective of this study.

#### 5.2.3. Results and discussion

The fictitious alumnus described in the questionnaire was rated as more moral (*t* = 10.47, *p* < 0.001, Cohens’ *d* = 2.55) when he/she was described as a moral model (*M* = 6.59, *SD* = 1.39) than when he/she was presented as someone with a habit of stealing (*M* = 3.41, *SD* = 1.09), demonstrating that the manipulation of the moral reputation of the object was effective.

The analysis of the participants’ willingness to donate revealed that the participants in the moral reputation condition had a higher donation willingness than the participants in the condition of immoral reputation (87.18% vs. 59.38%, *χ*^2^ = 7.18, *df* = 1, *p* = 0.007). There existed no significant difference in positive emotion (*t* = 0.02, *p* = 0.988, Cohens’ *d* = 0.003) between the conditions of moral (*M* = 2.76, *SD* = 0.93) and immoral reputation (*M* = 2.76, *SD* = 0.79). Similarly, there was no significant difference (*t* = 1.07, *p* = 0.290, Cohens’ *d* = 0.26) in negative emotion between the participants in the condition of moral reputation (*M* = 2.50, *SD* = 0.97) and those in the condition of immoral reputation (*M* = 2.28, *SD* = 0.74).

Overall, *Hypotheses 2* and *4* are supported by combining the results of Study 2a and Study 2b. Moral people can obtain more help from others, and moral reputation mediates the positive effect of vertical spatial position and help received from others. Although the experimental-causal-chain design could examine the causal relationships between independent variable, mediator, and dependent variable, they were not in a single sample (Spencer et al., 2005). As a result, whether these relationships could be replicated in one sample need to be further explored. In addition, the experimental-causal-chain design cannot estimate the effect size of the mediating effect (Spencer et al., 2005). As the mediator in our model could also be measured, we employed the measurement-of-mediation design to cross-validate our theoretical model in Study 3.

## 6. Study 3

### 6.1. Participants

In the current work, we need to test the results of Study 1 and Study 2. Thus, the smallest effect size is chosen in Study 2b, which is Cohen’s *d* = 0.81. With the use of the G*Power (Faul et al., 2007), we calculated the necessary samples to carry out an experiment of a single-factor design with two levels (Effect Size *d* = 0.81, Type I error = 0.05, Statistical Power = 0.80). The recommended sample size in each group is 25. Thus, a total of 86 undergraduates from a university in southern China (42 males, 44 females, *M_age_* = 20.27, *SD* = 1.40) were recruited to participate in this experiment. In addition, they completed the experiment in the same laboratory.

### 6.2. Procedures

The experiment design is a single factor design with two levels (low position vs. high position). The 86 participants were randomly assigned to one of two conditions. Moreover, the participant was told that the researchers were investigators from the university alliance of the city, and they were commissioned by the alliance committee to investigate the donation behavior of the college students in this city. Subsequently, the researchers distributed questionnaires to the participants. The questionnaire included measures of the perceived morality of the donor recipients, willingness to donate, donation amount, and the participants’ emotions. Next, the researchers projected a portrait of the donor recipient (claimed to be a low-income undergraduate) and a basic introduction to the donor recipient on a big screen in the laboratory. The lab and photo were the same as those in Study 1. Besides, the descriptions were consistent across the two conditions. The only difference was that the participants in the condition of high position saw the portrait on the upper part of the screen, while the participants in the condition of low position saw the portrait on the lower part of the screen. After reading the information displayed on the screen, the participants filled in the questionnaires. Finally, the researchers debriefed the participants. In addition, no participant guessed the true objective of this study accurately.

### 6.3. The moral reputation of the donor recipient

The participants were invited to answer the following three questions on a nine-point scale from 1 (very immoral/unkind/dishonest) to 9 (very moral/kind/honest): (1) “What do you think of the donor recipient’s moral level in the photos?” (2) “What do you think of the donor recipient’s kindness level in the photos?” and (3) “What do you think of the donor recipient’s honesty level in the photos?” The scale exhibited good reliability for this measurement (*α* = 0.94).

### 6.4. Helping behavior

To measure the helping behavior of the participants, both the willingness to donate and the donation amount of the participants were investigated. A seven-point scale, from 1 (not at all) to 7 (very much) was used to measure the willingness to donate, using the question below: “To what extent are you willing to help this low-income undergraduate?” The donation amount was measured using the questions below: “If you have 200 RMB pocket money (about 33 U.S. dollars when the experiment was conducted), how much would you donate to help this low-income undergraduate?” And the participants were required to write their answer from 0 to 200.

### 6.5. Participants’ emotion

The participants indicated how they felt by answering the 20-item Positive and Negative Affectivity Scale (PANAS; Watson et al., 1988). The PANAS captured both positive affect (*α* = 0.88) and negative affect (*α* = 0.92) on a five-point scale (1 = very slightly or not at all, 5 = extremely).

### 6.6. Results and discussion

**Figure 4:**
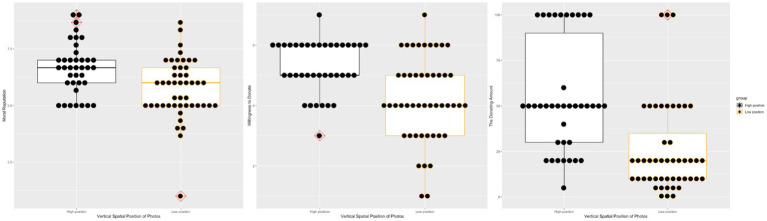
Two groups’ ratings about the donor recipient’s morality, willingness to donate, and the donation amount in Study 3.

The donor recipient was rated as more moral (*t* = 3.45, *p* < 0.001, Cohens’ *d* = 0.76) when his/her photo was projected on the upper part of the screen (*M* = 6.67, *SD* = 1.14) rather than the lower part of the screen (*M* = 5.74, *SD* = 1.31).^1^ Comparatively, the participants were more willing to help the donor recipient when his/her photo was projected on the upper part of the screen rather than the lower of the screen (*M_upper_* = 5.29, *SD* = 0.87; *M_bottom_* = 4.23, *SD* = 1.39; *t* = 4.33, *p* < 0.001, Cohens’ *d* = 0.92). Besides, the participants were also willing to donate more money to the donor recipient when his/her photo was projected on the upper part of the screen rather than the bottom part of the screen (*M_upper_* = 54.87, SD = 30.24; *M_bottom_* = 25.54, *SD* = 25.10; *t* = 4.92, *p* < 0.001, Cohens’ *d* = 1.06). Figure 4 presents the boxplot of these two groups’ ratings about the donor recipient’s morality, willingness to donate, and the donation amount.

We also performed a mediation analysis that revealed the mediating effects of moral reputation on the relationship between vertical spatial position and the willingness to donate/donation amount. Using model 4 of PROCESS macro (Hayes, 2013) with the Bootstrap set to 5,000, we find significant mediating effects of moral reputation on the relationships between vertical spatial position and the willingness to donate (indirect effect = 0.48, *SE* = 0.17, 95% CI [0.18, 0.85]) and the donation amount (indirect effect = 8.25, *SE* = 3.18, 95% CI [2.45, 14.74]). The obtained results supported *Hypothesis 1*, *Hypothesis 2*, *Hypothesis 3*, and *Hypothesis 4*.

In the current work, we cross-validate the results of Study 1 and Study 2 by using different scenarios. Then, we measure the moral reputation and the helping behavior in this study, allowing us to do a measurement-of-mediation design to test the mediating effects of moral reputation on the relationship between vertical spatial position and helping behavior. The results of the measurement-of-mediation design can also cross-validate the results of experimental-causal-chain design in Study 2.

## 7. General discussion

The results of Study 1 indicated that social targets’ vertical spatial position was positively associated with others’ helping behavior toward them. Study 2 explored the mediating mechanism between social targets’ vertical spatial position and others’ helping behavior with the experimental-causal-chain design. Specifically, Study 2a found a positive relationship between social targets’ vertical spatial position and their moral reputation. Study 2b demonstrated that social targets’ moral reputation could positively influence others’ helping behavior toward them. In general, Study 2 indicated that social targets’ moral reputation mediated the effect of social targets’ vertical spatial position on the help they received from others. Study 3 cross-validated the results of Study 2 by using the measurement-of-mediation design, which also revealed the mediating effects of moral reputation on the relationship between vertical spatial position and helping behavior. As a result, these results contribute to the literature of prosocial behavior and SCCB in several ways.

### 7.1. Theoretical implications

First, this study expands the antecedents of helping behavior by emphasizing the importance of help recipients’ factors, while previous studies mainly focused on the helpers’ traits and environmental characteristics in influencing helping behavior. Previous studies on helping behaviors have examined the antecedents from a variety of perspectives, such as the helpers’ personality (King et al., 2005), the helpers’ personal values (Shao et al., 2011), group cohesion (Rutkowski et al., 1983), and culture (Farh et al., 2004). However, few studies have focused on the characteristics of the recipients of helping behavior to examine why and how they can obtain help from others. It appears that prior studies tended to view help recipients as the passive objectives in the helping behavior (Bamberger, 2009; Thompson and Bolino, 2018). Moreover, our findings provide a new angle for understanding the occurrence of helping behavior, i.e., the recipients’ characteristics may also influence the occurrence of helping behavior. The current work found that the vertical spatial position of the help recipients could influence the occurrence of helping behavior.

Second, this study extends the metaphor connection between vertical spatial position and morality from conceptual cognition level to action-relevant outcomes. Previous studies have revealed only the metaphor connection between vertical spatial position and morality-related words by testing participants’ response time and accuracy of recognition (e.g., Meier et al., 2007a). Meanwhile, few studies have examined whether there are action-relevant outcomes of vertical spatial metaphor (Meier et al., 2012). In addition, this study found that vertical spatial position could influence moral judgement and helping behavior, which contributes to the literature on the connection between vertical spatial metaphor and morality by revealing action-relevant outcomes. The results of this study could also inspire researchers who concentrate on other cognitive metaphor topics, such as power and size (Schubert et al., 2009). Other cognitive metaphor topics could also expand their metaphor effects on specific behaviors.

Third, this study directly tests some predictions of the SCCB theory as well as extends the SCCB theory. The SCCB theory predicts that the higher social targets are on the chain of being, the more likely they will be positively treated by others; whereas the lower social targets are on the chain of being, the more likely they will be negatively treated by others (Brandt and Reyna, 2011). The results of this study support this proposition by revealing that the vertical spatial position and moral reputation are positively related to the helping behavior. Besides, the SCCB theory also points out that individuals use the vertical chain to understand morality. However, will the vertical spatial information of social targets directly influence others’ moral perception of the social targets? By combining the heuristic perspective, we found evidence to support this inference by demonstrating that the vertical spatial position could directly influence the social target’s moral reputation, which in turn can change their chain of being. This fact may have extended the theoretical boundary of SCCB.

Finally, the results of this study support the social adaptation function of morality (Haidt, 2013). Previous studies mainly focused on how and when individuals’ morality could lead them to help others (Sheng, 1994). However, this study found that moral reputation could be an important reason for others to offer help to the recipients. This result indicated that moral individuals not only do more prosocial behaviors themselves, but also can receive more prosocial behaviors from others.

### 7.2. Limitations and future directions

Nevertheless, there still exist limitations in this study. First, all the experiments in our studies were conducted among students in labs. Students usually lack social and work experiences, and laboratory experiments are different from real-life scenarios. As a result, the external validity of the study might require further demonstration. However, as the studies focused on a basic cognition process that is universal for most individuals, the student sample may not be a great concern (Randall and Gibson, 1990). It’s without a doubt that it will be meaningful to cross-validate the results by inviting participants who have more social and work experiences to take part in field studies.

Second, the measurement of helping behavior is essentially about assessing of behavioral intention rather than true behavior. The researchers measured individuals’ helping behavior by asking their willingness to help or the length of time they would be willing to help. In line with the theory of planned behavior, intention is the most important antecedent of behavior. However, it does not necessarily lead to the corresponding behavior (Ajzen, 1991). To enhance the linkage between helping intention and helping behavior, the researchers asked the participants to provide their telephone number for further contact to fulfill their offers of help in Study 1. All the participants who intended to help left their mobile phone numbers. This implied that there may exist a strong correlation between an intention and a behavior because the researchers had the opportunity to invite those participants to fulfill their promises. However, it is also necessary for future researchers to further verify, clarify, and extend the results of this study.

Third, due to the absence of a control condition in this study, the results only showed that social targets at a high vertical spatial position have a better moral reputation than people on a low position. The following question remains unanswered. Should the effect be attributed to individuals perceiving social targets on a high position as more moral than social targets on a middle position, or to individuals perceiving social targets on a low position as more immoral than social targets at a middle position? Regarding a control condition, our consideration was based on the reality that missing persons, commendations, and wanted notices usually have portraits placed on the upper or the bottom position in Chinese culture—there are few cases with portraits placed on the middle of such notices. As a result, if the portraits were put on the middle in the control condition, the participants might feel strange, which would influence the study’s validity by introducing a demanding effect. However, it is imperative to verify this effect, as there exist some conflicting results. For example, previous researchers found that “high is moral” and “down is immoral” (Meier et al., 2007a), whereas others supported only the former finding (Hill and Lapsley, 2009). As a result, a control group should be designed to clarify this in the future.

## Data availability statement

The raw data supporting the conclusions of this article will be made available by the authors, without undue reservation.

## Ethics statement

The studies involving human participants were reviewed and approved by Academic Committee of Jiangxi University of Finance and Economics. Written informed consent for participation was not required for this study in accordance with the national legislation and the institutional requirements.

## Author contributions

XM, JF, and JY designed this study. JF and JY collected data. XM analyzed the data and wrote the manuscript. All authors contributed to the article and approved the submitted version.

## Funding

This research was sponsored by the National Natural Science Foundation of China (grant nos. 72101103 and 72062017), the Bidding Project of Humanities and Social Sciences Key Research Base in Jiangxi Colleges and Universities (grant no. JD21047), and Social Science Foundation of Jiangxi Province (grant no. 20FX05).

## Conflict of interest

The authors declare that the research was conducted in the absence of any commercial or financial relationships that could be construed as a potential conflict of interest.

## Publisher’s note

All claims expressed in this article are solely those of the authors and do not necessarily represent those of their affiliated organizations, or those of the publisher, the editors and the reviewers. Any product that may be evaluated in this article, or claim that may be made by its manufacturer, is not guaranteed or endorsed by the publisher.

## Footnotes

^1^One of the total 86 participants failed to answer the question about the moral reputation of the donor recipient.
